# Supplementary material for: Resolution of Resilience: Empirical Findings on the Challenges Faced and the Mitigation Strategies Adopted by Community Health Workers (CHWs) to Provide Maternal and Child Health (MCH) Services during the COVID-19 Pandemic in the Context of Odisha, India
Source: Healthcare (Basel). 2022 Jan 3;10(1):88. doi: 10.3390/healthcare10010088 (PMC8775981; doi:10.3390/healthcare10010088)
Supplement: Supplementary file 1 [file healthcare-10-00088-s001.zip › healthcare-1374434-supplementary.pdf]

healthcare

MDPI

Supplementary File S1

Themes	Challenges in Delivering MCH Services			Strategies to Overcome the Challenges			Role in District COVID Activities	
Categories	Personal	Facility	Community	Personal	Facility level	Community	COVID related work	Services for migrant population
Sub-categories	Lack of family support<br><br>Over burden of work<br><br>Fear of contracting infection	Depriving ASHA as a birth companion<br><br>Disrespectful behaviour<br><br>Transportation service<br><br>Lack of PPE	Lack of community support<br><br>Stigma<br><br>Violence	Counseling	Transportati on<br><br>Financial support	Awareness	Testing<br><br>Reporting<br><br>Follow up	Testing<br><br>Delivery services<br><br>Immunization
Codes	Violence, stigma, lack of PPE, lack of training, lack of community support			Share with family, awareness, home visits,			Reporting, testing, IEC activity, follow up visits,	
